# Epidemiology of severe acute respiratory infections from hospital-based surveillance in Madagascar, November 2010 to July 2013

**DOI:** 10.1371/journal.pone.0205124

**Published:** 2018-11-21

**Authors:** Norosoa Harline Razanajatovo, Julia Guillebaud, Aina Harimanana, Soatiana Rajatonirina, Elisoa Hariniaina Ratsima, Zo Zafitsara Andrianirina, Hervé Rakotoariniaina, Todisoa Andriatahina, Arnaud Orelle, Rila Ratovoson, Judickaelle Irinantenaina, Dina Arinalina Rakotonanahary, Lovasoa Ramparany, Frédérique Randrianirina, Vincent Richard, Jean-Michel Heraud

**Affiliations:** 1 National Influenza Centre, Virology Unit, Pasteur Institute of Madagascar, Antananarivo, Madagascar; 2 Epidemiology Unit, Pasteur Institute of Madagascar, Antananarivo, Madagascar; 3 Center for Biological Analysis, Pasteur Institute of Madagascar, Antananarivo, Madagascar; 4 Soavinandriana Hospital, Antananarivo, Madagascar; 5 Secondary Level Public Hospital, Moramanga, Madagascar; Defense Threat Reduction Agency, UNITED STATES

## Abstract

**Background:**

Few comprehensive data exist regarding the epidemiology of severe acute respiratory infections (SARI) in low income countries. This study aimed at identifying etiologies and describing clinical features of SARI-associated hospitalization in Madagascar.

**Methods:**

It is a prospective surveillance of SARI in 2 hospitals for 3 years. Nasopharyngeal swabs, sputum, and blood were collected from SARI patients enrolled and tested for viruses and bacteria. Epidemiological and clinical information were obtained from case report forms.

**Results:**

Overall, 876 patients were enrolled in the study, of which 83.1% (728/876) were tested positive for at least one pathogen. Viral and bacterial infections occurred in 76.1% (667/876) and 35.8% (314/876) of tested samples, respectively. Among all detected viruses, respiratory syncytial virus (RSV) was the most common (37.7%; 348/924) followed by influenza virus A (FLUA, 18.4%; 170/924), rhinovirus (RV, 13.5%; 125/924), and adenovirus (ADV, 8.3%; 77/924). Among bacteria, *Streptococcus pneumoniae* (*S*. *pneumoniae*, 50.3%, 189/370) was the most detected followed by *Haemophilus influenzae* type b (Hib, 21.4%; 79/370), and Klebsiella (4.6%; 17/370). Other Streptococcus species were found in 8.1% (30/370) of samples. Compared to patients aged less than 5 years, older age groups were significantly less infected with RSV. On the other hand, patients aged more than 64 years (OR = 3.66) were at higher risk to be infected with FLUA, while those aged 15–29 years (OR = 3.22) and 30–64 years (OR = 2.39) were more likely to be infected with FLUB (influenza virus B).

**Conclusion:**

The frequency of influenza viruses detected among SARI patients aged 65 years and more highlights the need for health authorities to develop strategies to reduce morbidity amongst at-risk population through vaccine recommendation. Amongst young children, the demonstrated burden of RSV should guide clinicians for a better case management of children. These findings reveal the need to develop point-of-care tests to avoid overuse of antibiotics and to promote vaccine that could reduce drastically the RSV hospitalizations.

## Background

Severe acute respiratory infections (SARI) are among the leading cause of hospitalization and deaths worldwide [1]. It is estimated that about 4.2 million of deaths are attributed to SARI annually [2]. Estimation in 2010 have shown that 11 million of children aged less than 5 years were admitted for SARI in developing countries, with an estimated case fatality ratio of 2.3%, compared to 570 000 cases and a case fatality ratio of 0.6% in developed countries [3]. In low income countries, the annual incidence of pneumonia is estimated at 151 million children of whom 13% are severe enough to warrant hospitalization [1, 4]. Nearly 2 million children die from pneumonia every year, 70% of which occur in Africa and Southeast Asia [5, 6].

SARI may be caused by various pathogens. While bacterial infections play a critical role in causing life-threatening pneumonia [1, 7–9], viral infections are associated with significant proportion that range from mild to severe infections [10, 11]. However, due to the lack of gold standard methods to rapidly differentiate between viral and bacterial infections, most of the patients may be treated empirically with antibiotics [12]. Rapid etiologic diagnosis is therefore needed to select the most appropriate treatment protocol and to avoid the development of bacterial resistance.

Following the A/H1N1/2009 influenza pandemic that was associated with a high morbidity and an increased risk of mortality among particular groups [13], a number of countries have strengthened vigilance for the surveillance of severe diseases and deaths in order to rapidly detect new viruses and to provide information in assessing the impact on the population and having operational preparedness plans. So far, only few sub-Saharan African countries are collecting data on hospitalization associated to acute respiratory illness [14–16]. In Madagascar, the sentinel syndromic surveillance has been collecting SARI data since 2010. However, hospitals only collect few data that are not exploitable for analysis. Thus, data are still scarce regarding the epidemiology of SARI in this country. During the last influenza pandemic in 2009, an excess of mortality was observed among inhabitant of the main capital city of Antananarivo [17]. Following the pandemic, health authorities requested to develop an active surveillance of SARI in order to better understand the epidemiology of this disease in the context of Madagascar. The present study aimed to identify etiologies of SARI and to assess clinical features of hospitalization linked to SARI in 2 hospitals during 3 years of surveillance.

## Material and methods

### Study sites

A prospective hospital-based SARI surveillance was conducted from November 2010 to July 2013 in 2 selected sites: the Soavinandriana hospital of Antananarivo and the secondary level public hospital (CHD II) of Moramanga. The CENHOSOA is a national referral hospital and is among the 4 largest hospitals that deserve Antananarivo, the main capital city of Madagascar which has around 2.5 million inhabitants. The CHD II in Moramanga is the only local referral hospital for the health district of Moramanga that has 250 000 inhabitants. It is located 115 km east from the capital city. Antananarivo and Moramanga present the same climatic profile: hot and rainy in summer and cold and dry in winter, but Moramanga encompasses both semi-urban and rural areas.

### Study subjects

All patients presenting SARI symptoms at admission were enrolled. SARI case definition was defined according to the WHO case definition as fever (T≥38°C) or history of fever and cough that required hospitalization. For children less than 5 years, the eligibility criteria were suspected sepsis or SARI diagnosed by physician including bronchiolitis, pneumonia, bronchitis, pleural effusion, and cough or difficult breathing as previously published [18]. The onset of illness should be less than 7 days before hospitalization. For each consented patient, demographic, socio-economic, clinical, and epidemiological data were recorded in case report forms (CRFs).

### Biological analysis

Nasopharyngeal, blood, and sputum specimens were collected for each patient enrolled from Monday to Friday and shipped the same day to laboratories located at the Pasteur Institute of Madagascar where they were immediately processed or stored at 4°C until testing (test were performed within 48 hours post-sampling). All procedures for the biological analyses have been previously described [18, 19]. Briefly, nasopharyngeal swabs were screened for 14 respiratory viruses using in-house multiplex real-time PCR [19]. Sputum was collected for cytobacteriologic testing while blood samples were used for cell blood count.

### Data analysis

Single infection was defined as an infection caused by one pathogen (virus or bacteria) and multiple infection as an infection caused by at least 2 pathogens (virus/virus, virus/bacteria or bacteria/bacteria) in a single sample. Patients were divided into 5 age groups: infants and young children (≤ 5 years old); children (5–14 years old); adolescent and young adults (15–29 years old); adults (30–64), and elderly (≥ 65 years old). Demographic, clinical characteristics, and etiologies were compared by study sites and by age groups. Univariate analysis and logistic regression were performed using R software. In univariate analysis, qualitative variables were compared with Fisher’s exact test and quantitative variables by non-parametric tests (Wilcoxon test). Furthermore, logistic regressions were performed to adjust Odds-Ratio (OR) found via maximum-likelihood estimation according to age group for the different variables as dependent variables and to compare each of them according to age group by Wald test. The age group less than 5 years has been considered as reference group. Statistical differences were considered significant for two-sided p-values < = 0.05.

### Ethics statement

Although SARI surveillance in Madagascar is not considered as research, this prospective study requested extra sampling and the collection of personal information. For that reason, the present study was submitted and approved by the national ethics committee from the ministry of health in Madagascar (Authorization N°068-MSANP/CE). Adult participants or parents were fully informed of the study’s objectives and procedures. Written informed consent was obtained from patients enrolled in the study. Young participants aged from 7 to 18 years old were asked to sign an informed assent form if they were willing to take part in the study. For all patients aged less than 18 years, consent was obtained from the parents or legal guardians. After obtaining the consent/assent form, the team survey proceeded to collect samples and fill out the CRFs. Refused consent and prior hospitalization within 2 weeks before consultation were reasons for exclusion.

## Results

### Patient’s demographic characteristics

From November 2010 to July 2013, 876 hospitalized patients completed the established SARI case definition of whom 657 cases (75.0%) were from CENHOSOA and 219 cases (25.0%) from CHD II Moramanga. The mean age was 8.2 years ranging from 1 day to 89 years and 54.8% of patients were male cases. The majority of recruited patients were children aged less than 5 years and accounting for 81% of total inclusion. Adult aged more than 65 years represented 2% of cases (Table 1). Differences of mean age (2.8 years vs. 9.9 years; p<0.001; Wilcoxon test) and age repartition (p<0.001) of included patients were observed between the 2 study sites with patients hospitalized in CHD II Moramanga being younger than those hospitalized in CENHOSOA (Table 1).

**Table 1:** Demographic and clinical characteristics of patients hospitalized for SARI, Madagascar, November 2010 to July 2013.

Variables		Global (N = 876)		Antananarivo (N = 657)		Moramanga (N = 219)	p-value
**Age**							
mean [95%CI]		8.2 [7.0–9.3]		9.9 [8.5–11.4]		2.8 [1.5–4.1]	<0.001
**Age groups**		**n (%)**		**n (%)**		**n (%)**	
< 5yrs		710 (81.1)		502 (76.4)		208 (95.0)	<0.001
5-14yrs		37 (4.2)		33 (5.0)		4 (1.8)	
15-29yrs		26 (3.0)		26 (4.0)		0 (0.0)	
30-64yrs		84 (9.6)		79 (12.0)		5 (2.3)	
> = 65yrs		19 (2.2)		17 (2.6)		2 (0.9)	
**Gender**		**n (%)**		**n (%)**		**n (%)**	
Female		396 (45.2)		295 (44.9)		101 (46.1)	0.8
Male		480 (54.8)		362 (55.1)		118 (53.9)	
**Symptoms**	***n***	***Presence (%)***	***n***	***Presence (%)***	***n***	***Presence (%)***	
Fever	*860*	590 (68.6)	*651*	445 (68.4)	*209*	145 (69.4)	0.8
Dry cough	*857*	411 (48.0)	*647*	296 (45.7)	*210*	115 (54.8)	0.03
Productive cough	*857*	454 (53.0)	*647*	357 (55.2)	*210*	97 (46.2)	0.03
Dyspnea	*860*	737 (85.7)	*648*	564 (87.0)	*212*	173 (81.6)	0.06
Chest pain	*751*	110 (14.6)	*615*	96 (15.6)	*136*	14 (10.3)	0.1
Runny nose	*858*	598 (69.7)	*646*	419 (64.9)	*212*	179 (84.4)	<0.001
Sore throat	*783*	95 (12.1)	*626*	89 (14.2)	*157*	6 (3.8)	<0.001
Headache	*753*	107 (30.3)	*619*	97 (15.7)	*134*	10 (7.5)	0.01
Thrill	*851*	145 (17.0)	*643*	125 (19.4)	*208*	20 (9.6)	0.001
Sweats	*856*	259 (30.0)	*646*	188 (29.1)	*210*	71 (33.8)	0.2
Anorexia	*859*	432 (50.3)	*649*	306 (47.1)	*210*	126 (60.0)	0.001
Vomiting	*861*	191 (22.2)	*650*	142 (21.8)	*211*	49 (23.2)	0.7
Diarrhea	*859*	112 (13.0)	*650*	85 (13.1)	*209*	27 (12.9)	1
Weight loss	*853*	245 (28.7)	*643*	174 (27.1)	*210*	71 (33.8)	0.07
Asthenia	*854*	422 (49.4)	*643*	297 (46.2)	*211*	125 (59.2)	0.001
GPD	*859*	227 (26.4)	*648*	161 (24.8)	*211*	66 (31.3)	0.07
Inter recess	*855*	598 (69.9)	*645*	445 (69.0)	*210*	153 (72.9)	0.3
MNW	*854*	446 (52.2)	*646*	334 (51.7)	*208*	112 (53.8)	0.6
Cyanosis	*848*	141 (16.6)	*642*	133 (20.7)	*206*	8 (3.9)	<0.001

GPD: General physical deterioration; Inter recess: Intercostal recession; MNW: Movement of nose wings.

n = number of patients that responded by “yes” or “no” for a given symptom.

Statistical analyses were performed using Fisher’s exact test.

### Clinical characteristics

With exception of dyspnea and fever which are among the principal inclusion criteria, runny nose (69.7%), intercostal recessions (69.9%), productive cough (53.0%), movement of nose wings (52.6%), and anorexia (50.3%) were the most recorded at the time of examination. Bronchiolitis (44.5%) was the most clinical diagnostic made by clinicians at admission followed by bronchopneumonia (15.0%) and pneumonia (8.8%). Other low respiratory tract infections (bronchoalveolitis/exacerbation of chronic obstructive pulmonary disease (COPD), pleuropneumonia, and acute lobar pneumonia) were observed in 14.8% of inpatients (S1 Table). Comparison of clinical symptoms between the 2 sites showed that dry cough (p = 0.026), asthenia (p = 0.001), runny nose (p<0.001), and anorexia (p = 0.001) were statistically frequently observed in patients hospitalized in CHDII Moramanga whereas productive cough (p = 0.026), sore throat (p<0.001), headache (p = 0.014), thrill (p = 0.001), and cyanosis (p<0.001) were significantly associated with patients hospitalized in CENHOSOA (Table 1).

### Infections

At least one pathogen was found in 83.1% (728/876) of tested patients of which 38.8% (340/876) were single infection and 44.3% (388/876) were multiple infections. The highest rate of infection was obtained in patients aged less than 5 years (85.2%; 605/710). For this age group, single and multiple infections were reported in 79.1% (269/340) and 86.6% (336/388) of cases, respectively. The type of infection (mono-infection vs. multiple infection) differed significantly between age groups (p = 0.03, Fisher’s exact test). Indeed, patients aged 30–64 years were significantly less likely at risk for being co-infected (OR = 0.4; p = 0.003) compared to those aged less than 5 years (S2 Table).

### Etiologies

Overall, viral and bacterial infections occurred in 76.1% (667/876) and 35.8% (314/876) of tested samples, respectively. Among the total number of detected viruses (N = 924), respiratory syncytial virus (RSV) was the most commonly identified (37.7%; 348/924) followed by influenza virus A (FLUA, 18.4%; 170/924), rhinovirus (RV, 13.5%; 125/924), adenovirus (ADV, 8.3%; 77/924), influenza virus B (FLUB, 6.3%; 58/924), bocavirus (BOV, 4.3%; 40/924), and human metapneumovirus (HMPV, 3.6%; 33/924) (Table 2). The representativeness of other respiratory viruses ranged from 2.3% to 0.2% of infection. Among the total detected bacteria (N = 370), *Streptococcus pneumoniae* (*S*. *pneumoniae*, 50.3%, 189/370) was the most diagnosed followed by *Haemophilus influenzae* type b (Hib, 21.4%; 79/370), other Streptococcus species (8.1%; 30/370), Klebsiella (4.6%; 17/370), Branhamella (4.1%; 15/370), and Enterobacteria (3.8%; 14/370). Other germs including atypical bacteria were detected in a proportion ranging from 2.7% to 0.3% of infection.

**Table 2:** Proportion of pathogens detected in SARI-associated hospitalization, Madagascar, November 2010 to July 2013.

**VIRUS**	**N = 924**	**%**
Respiratory syncytial virus	348	37.7
Influenza virus A	170	18.4
Rhinovirus	125	13.5
Adenovirus	77	8.3
Influenza virus B	58	6.3
Bocavirus	40	4.3
Human metapneumovirus	33	3.6
Coronavirus-OC43	21	2.3
Coronavirus-NL63	15	1.6
Parainfluenza virus 2	12	1.3
Parainfluenza virus 1	10	1.1
Parainfluenza virus 3	9	1.0
Coronavirus-229E	4	0.4
Coronavirus-HKU1	2	0.2
**BACTERIA**	**N = 370**	**%**
*Streptococcus pneumoniae*	186	50.3
*Haemophilus influenzae* type b	79	21.4
Streptococcus*	30	8.1
Klebsiella	17	4.6
Branhamella	16	4.3
Enterobacteria	14	3.8
*Staphylococcus aureus*	10	2.7
*Escherichia coli*	4	1.1
Acinetobacter	3	0.8
Aerococcus	3	0.8
Moraxella	2	0.5
*Pseudomonas aeruginosa*	2	0.5
Listeria	1	0.3
*Staphylococcus haemolyticus*	1	0.3
Stenotrophomonas	1	0.3
*Proteus mirabilis*	1	0.3
*Serratia marcescens*	1	0.3

* Other species of Streptococcus.

Comparison of the prevalence of pathogens by study sites revealed that FLUA (p = 0.04), *S*. *pneumoniae* (p = 0.003), and Hib (p = 0.04) were statistically relatively common in patients hospitalized in CENHOSOA (S3 Table). In respect to multiple infections, FLUA (OR = 4.3), RV (OR = 3.6), HMPV (OR = 3.3), ADV (OR = 4.0), BOV (OR = 5.2), *S*. *pneumoniae* (OR = 8.0), and Hib (OR = 7.9) were statistically more often found in association with other pathogens. The multiple infection involving RSV and *S*. *pneumoniae* was the most common (68 cases) followed by RSV and FLUA (54 cases), FLUA and *S*. *pneumoniae* (40 cases), RSV and RV (37 cases), *S*. *pneumoniae* and RV (36 cases), *S*. *pneumoniae* and Hib (35 cases), and RSV and Hib (31 cases). Surprisingly, 118 patients were co-infected with 4, 5, and 6 different pathogens (92, 20, and 6, respectively) (Table 3).

**Table 3:** Etiological agents and multiple infections among patients hospitalized for SARI, Madagascar, November 2010 to July 2013.

	**FLUA**	**FLUB**	**COV-OC43**	**COV-NL63**	**COV-229E**	**COV-HKU1**	**RSV**	**HMPV**	**RV**	**PIV1**	**PIV2**	**PIV3**	**ADV**	**BOV**	***S*. *pneumoniae***	**Hib**	**Strepto**
**FLUA**	**47**	17	3	1	1	0	54	6	17	0	0	2	12	8	40	17	10
**FLUB**	-	**22**	0	2	0	0	12	1	5	0	0	0	4	4	16	6	2
**COV-OC43**	-	-	**3**	1	0	0	7	1	2	0	3	0	2	2	6	0	1
**COV-NL63**	-	-	-	**3**	0	0	6	0	3	0	0	0	1	1	5	2	0
**COV-229E**	-	-	-	-	**2**	0	1	0	0	0	0	0	0	0	0	0	0
**COV-HKU1**	-	-	-	-	-	**1**	0	0	1	0	0	0	0	0	0	0	0
**RSV**	-	-	-	-	-	-	**171**	3	37	1	1	0	25	15	68	31	7
**HMPV**	-	-	-	-	-	-		**9**	2	2	1	0	0	0	12	5	1
**RV**	-	-	-	-	-	-	-	-	**33**	1	3	1	16	13	36	11	3
**PIV1**	-	-	-	-	-	-	-	-	-	**2**	0	0	2	1	2	2	2
**PIV2**	-	-	-	-	-	-	-	-	-	-	**4**	0	0	1	2	2	1
**PIV3**	-	-	-	-	-	-	-	-	-	-	-	**2**	0	1	3	2	0
**ADV**	-	-	-	-	-	-	-	-	-	-	-	-	**17**	7	20	6	4
**BOV**	-	-	-	-	-	-	-	-	-	-	-	-	-	**6**	13	4	0
***S*. *pneumoniae***	-	-	-	-	-	-	-	-	-	-	-	-	-	-	**29**	35	0
**Hib**	-	-	-	-	-	-	-	-	-	-	-	-	-	-	-	**11**	3
Strepto*																	**6**
**Monoinfection**	47	22	3	3	2	1	171	9	33	2	4	2	17	6	29	11	6
**2 pathogens**	73	15	11	6	2	1	110	14	50	4	3	6	35	11	84	26	15
**3 pathogens**	38	13	5	3	0	0	47	10	27	3	4	0	15	14	51	29	8
**4 pathogens**	10	5	1	2	0	0	16	0	13	1	1	1	7	6	17	11	1
**5 pathogens**	1	2	1	1	0	0	4	0	2	0	0	0	2	2	4	1	0
**6 pathogens**	1	1	0	0	0	0	0	0	0	0	0	0	1	1	1	1	0
**Total**	**170**	**58**	**21**	**15**	**4**	**2**	**348**	**33**	**125**	**10**	**12**	**9**	**77**	**40**	**186**	**79**	**30**

FLUA: Influenza virus A; FLUB: Influenza virus B; COV: Coronavirus; RSV: Respiratory syncytial virus; HMPV: Human metapneumovirus; RV: Rhinovirus; PIV: Parainfluenza virus; ADV: Adenovirus; BOV: Bocavirus; *S*. *pneumoniae*: *Streptococcus pneumoniae*; Hib: *Haemophilus influenzae* type b; Strepto: Streptococcus.

*: other species of Streptococcus.

#### Comparison of demographic and clinical characteristics of SARI patients by age group

Analysis of data by the age group showed that the frequency of clinical symptoms significantly varied across groups. Indeed, by multivariate analysis adjusted by age, patients aged ≥5 years appeared to suffer chest pain compared to patients aged <5 years while dry cough and GPD were more often observed in the elderly. Dyspnea and cyanosis were significantly more common in adults aged 30–64 years. Headache, thrill, sweats, anorexia, weight loss, and asthenia were more often observed in the older age groups (15–29 years, 30–64 years and ≥65 years). Sore throat was frequently reported in the age groups 5–14 years, 15–29 years and 30–64 years. Runny nose and intercostal recession appeared to be relatively infrequent in the older age groups (15–29 years, 30–64 years and ≥65 years) (Table 4 and S4 Table).

**Table 4:** Results of logistic regression for demographic and clinical characteristics of SARI patients adjusted by age group, Madagascar, November 2010 to July 2013.

Variables		< 5yrs (N = 710)	5-14yrs (N = 37)	15-29yrs (N = 26)	30-64yrs (N = 84)	>= 65yrs (N = 19)
**Gender**	**N = 876**	**n (%)**	**n (%)**	**n (%)**	**n (%)**	**n (%)**
Female	*396*	325 (82.1)	17 (4.3)	11 (2.8)	34 (8.6)	9 (2.3)
Male	*480*	385 (80.2)	20 (4.2)	15 (3.1)	50 (10.4)	10 (2.1)
	*OR [95%CI]*	1	0.99 [0.5–1.9]	1.2 [0.5–2.6]	1.2 [0.8–2.0]	0.9 [0.4–2.4]
	p-value**	--	0.9	0.7	0.4	0.9
Symptoms*						
Dry cough	***857***	321 (46.4)	14 (37.8)	15 (60.0)	46 (54.8)	15 (78.9)
	*OR [95%CI]*	1	0.7 [0.4–1.4]	1.7 [0.8–4.0]	1.4 [0.9–2.2]	4.3 [1.6–15.3]
	*p-value*	--	0.3	0.2	0.1	**0.01**
Dyspnea	***860***	585 (84.3)	32 (86.5)	23 (88.5)	80 (95.2)	17 (89.5)
	*OR [95%CI]*	1	1.2 [0.5–3.6]	1.4 [0.5–6.1]	3.7 [1.5–12.4]	1.6 [0.5–10.1]
	*p-value*	--	0.7	0.6	**0.01**	0.5
Chest pain	***751***	43 (7.3)	8 (22.2)	9 (34.6)	42 (50.6)	8 (42.1)
	*OR [95%CI]*	1	3.6 [1.5–8.1]	6.7 [2.7–15.6]	13 [7.6–22.2]	9.2 [3.4–24.0]
	*p-value*	--	**0.003**	**<0.001**	**<0.001**	**<0.001**
Runny nose	***858***	524 (75.7)	30 (81.1)	7 (26.9)	30 (35.7)	7 (36.8)
	*OR [95%CI]*	1	1.4 [0.6–3.5]	0.1 [0.1–0.3]	0.2 [0.1–0.3]	0.2 [0.1–0.5]
	*p-value*	--	0.459	**<0.001**	**<0.001**	**0.001**
Sore throat	***783***	58 (9.4)	10 (27.0)	7 (26.9)	16 (19.0)	4 (21.1)
	*OR [95%CI]*	1	3.6 [1.6–7.5]	3.6 [1.3–8.5]	2.3 [1.2–4.1]	2.6 [0.7–7.4]
	*p-value*	--	**0.001**	**0.006**	**0.008**	0.1
Headache	***753***	32 (5.4)	2 (5.7)	13 (50.0)	48 (57.8)	12 (63.2)
	*OR [95%CI]*	1	1.1 [0.2–3.7]	17.4 [7.4–41.1]	23.9 [13.42.5]	29 [11.3–85.3]
	*p-value*	--	0.9	**<0.001**	**<0.001**	**<0.001**
Thrill	***851***	62 (9.0)	6 (16.7)	15 (57.7)	50 (59.5)	12 (63.2)
	*OR [95%CI]*	1	2 [0.7–4.7]	13.7 [6.1–31.9]	14.8 [9.0–24.8]	17.3 [6.7–47.9]
	*p-value*	--	0.1	**<0.001**	**<0.001**	**<0.001**
Sweats	***856***	161 (23.3)	11 (29.7)	19 (73.1)	54 (64.3)	14 (73.7)
	*OR [95%CI]*	1	1.4 [0.7–2.8]	8.9 [3.8–23.2]	5.9 [3.7–10.0]	9.2 [3.5–28.8]
	*p-value*	--	0.4	**<0.001**	**<0.001**	**<0.001**
Anorexia	***859***	325 (46.9)	20 (54.1)	18 (69.2)	52 (61.9)	17 (89.5)
	*OR [95%CI]*	1	1.3 [0.7–2.6]	2.6 [1.1–6.3]	1.8 [1.2–3.0]	9.6 [2.7–61.0]
	*p-value*	--	0.4	0.03	**0.01**	**0.003**
Weight loss	***853***	171 (24.9)	12 (32.4)	16 (61.5)	34 (40.5)	12 (63.2)
	*OR [95%CI]*	1	1.5 [0.7–2.9]	4.8 [2.2–11.2]	2.1 [1.3–3.3]	5.2 [2.1–14.1]
	*p-value*	--	0.3	**<0.001**	**0.003**	**0.001**
Asthenia	***854***	302 (43.9)	17 (45.9)	21 (80.8)	65 (77.4)	17 (89.5)
	*OR [95%CI]*	1	1.1 [0.6–2.1]	5.4 [2.2–16.2]	4.4 [2.6–7.6]	10.9 [3.1–68.9]
	*p-value*	--	0.8	**0.001**	**<0.001**	**0.002**
GPD	***859***	174 (25.1)	7 (18.9)	11 (42.3)	22 (26.2)	13 (68.4)
	*OR [95%CI]*	1	0.7 [0.3–1.5]	2.2 [1.0–4.8]	1.1 [0.6–1.8]	6.5 [2.5–18.6]
	*p-value*	--	0.4	0.05	0.8	**<0.001**
Inter recess	***855***	516 (74.7)	23 (62.2)	12 (46.2)	38 (46.3)	9 (47.4)
	*OR [95%CI]*	1	0.6 [0.3–1.1]	0.3 [0.1–0.6]	0.3 [0.2–0.5]	0.3 [0.1–0.8]
	*p-value*	--	0.1	**0.002**	**<0.001**	**0.011**
Cyanosis	***848***	101 (14.7)	3 (8.1)	6 (23.1)	26 (32.5)	5 (26.3)
	*OR [95%CI]*	1	0.5 [0.1–1.5]	1.7 [0.6–4.2]	2.8 [1.7–4.6]	2.1 [0.7–5.5]
	*p-value*	--	0.3	0.2	**<0.001**	0.2

GPD: General physical deterioration; Inter recess: Intercostal recession.

n = number of patients that responded “yes” for the presence of symptom.

* Only those which were statistically significance are shown.

** P-values that are significant are shown in bold. Statistical analyses were performed using Wald test. The age group less than 5 years was considered as reference group.

Regarding infection, prevalence of RSV (p<0.001) and influenza viruses (p<0.001) substantially varied depending the age group. As expected, RSV accounted for the highest proportion of SARI in young children (45.9%; 326/710). In fact, the older age groups were significantly less at risk to develop an RSV infection compared to the age group less than 5 years (Table 5). *S*. *pneumoniae* and influenza viruses accounted respectively for 22.4%; (159/710) and 20.4% (145/710) of infection. On the other hand, influenza viruses were the most predominant agents in patients aged more than 5 years (5–14 years, 15–29 years, 30–64 years, and ≥65 years), showing an increasing pattern of infection with the increasing age of patients. More accurately, patients aged between 30–64 years (OR = 2.4) and ≥65 years (OR = 3.7) were at higher risk to develop FLUA infection while patients aged between 15–29 years (OR = 3.2) and between 30–64 years (OR = 2.4) were more likely to catch FLUB infection. By multivariate analysis, the age group between 30–64 years were less likely to suffer from *S*. *pneumoniae* infection (OR = 0.5) than the age group less than 5 years (Table 5).

**Table 5:** Results of logistic regression for etiologic agents from SARI patients adjusted by age group, Madagascar, November 2010 to July 2013.

Pathogens		< 5yrs (N = 710)	5-14yrs (N = 37)	15-29yrs (N = 26)	30-64yrs (N = 84)	>= 65yrs (N = 19)
FLUA	*n (%)*	121 (17.0)	7 (18.9)	5 (19.2)	28 (33.3)	9 (47.4)
	*OR [95%CI]*	1	1.1 [0.5–2.5]	1.2 [0.4–2.9]	2.4 [1.5–4.0]	3.7 [1.5–8.9]
	*p-value*	--	0.8	0.8	**0.001**	**0.004**
FLUB	*n (%)*	38 (5.4)	4 (10.8)	4 (15.4)	10 (11.9)	2 (10.5)
	*OR [95%CI]*	1	2.1 [0.6–5.7]	3.2 [0.9–8.9]	2.4 [1.1–4.8]	2.1 [0.3–7.6]
	*p-value*	--	0.2	**0.04**	**0.02**	0.3
Influenza*	*n (%)*	145 (20.4)	11 (29.7)	9 (34.6)	36 (42.9)	10 (52.6)
	*OR [95%CI]*	1	1.7 [0.8–3.3]	2.1 [0.9–4.6]	2.9 [1.8–4.7]	4.3 [1.7–11.1]
	*p-value*	--	0.2	0.9	**<0.001**	**0.002**
COV-OC43	*n (%)*	19 (2.7)	2 (5.4)	0	0	0
	*OR [95%CI]*	1	2.1 [0.3–7.6]	0	0	0
	*p-value*	--	0.3	--	--	--
COV-NL63	*n (%)*	14 (2.0)	0	0	1 (1.2)	0
	*OR [95%CI]*	1	0	0	0.6 [0.03–3.04]	0
	*p-value*	--	--	--	0.6	--
RSV	*n (%)*	326 (45.9)	8 (21.6)	4 (15.4)	8 (9.5)	2 (10.5)
	*OR [95%CI]*	1	0.3 [0.1–0.7]	0.2 [0.1–0.6]	0.1 [0.1–0.3]	0.1 [0.02–0.5]
	*p-value*	--	**0.006**	**0.005**	**<0.001**	**0.009**
HMPV	*n (%)*	29 (4.1)	2 (5.4)	0	1 (1.2)	1 (5.3)
	*OR [95%CI]*	1	1.3 [0.2–4.7]	0	0.3 [0.02–1.4]	1.3 [0.8–6.7]
	*p-value*	--	0.7	--	0.2	0.8
Rhinovirus	*n (%)*	107 (15.1)	6 (16.2)	1 (3.8)	10 (11.9)	1 (5.3)
	*OR [95%CI]*	1	1.1 [0.4–2.5]	0.2 [0.01–1.1]	0.8 [0.4–1.5]	0.3 [0.02–1.5]
	*p-value*	--	0.9	0.1	0.4	0.3
Adenovirus	*n (%)*	69 (9.7)	3 (8.1)	2 (7.7)	3 (3.6)	0
	*OR [95%CI]*	1	0.8 [0.2–2.4]	0.8 [0.1–2.7]	0.3 [0.1–1.0]	0
	*p-value*	--	0.7	0.7	0.1	--
Bocavirus	*n (%)*	34 (4.8)	4 (10.8)	1 (3.8)	1 (1.2)	0
	*OR [95%CI]*	1	2.4 [0.7–6.5]	0.8 [0.04–3.9]	0.2 [0.01–1.1]	0
	*p-value*	--	0.1	0.8	0.2	--
*S*. *pneumoniae*	*n (%)*	159 (22.4)	10 (27.0)	4 (15.4)	10 (11.9)	3 (15.8)
	*OR [95%CI]*	1	1.3 [0.6–2.6]	0.6 [0.2–1.7]	0.5 [0.2–0.9]	0.7 [0.2–2.0]
	*p-value*	--	0.5	0.4	**0.03**	0.5
Hib	*n (%)*	70 (9.9)	3 (8.1)	0	4 (4.8)	2 (10.5)
	*OR [95%CI]*	1	0.8 [0.2–2.3]	0	0.5 [0.1–1.1]	1.1 [0.2–3.9]
	*p-value*	--	0.7	--	0.1	0.9

FLUA: Influenza virus A; FLUB: Influenza virus B; COV: Coronavirus; RSV: Respiratory syncytial virus; HMPV: Human metapneumovirus; *S*. *pneumoniae*: *Streptococcus pneumoniae*; Hib: *Haemophilus influenzae* type b.

n: number of patients who were positive for a pathogen.

* All influenza virus cases.

** P-values that are significant are shown in bold. Statistical analyses were performed using Wald test. The age group less than 5 years was considered as reference group.

#### Pattern of circulation of respiratory pathogens

**Fig 1:**
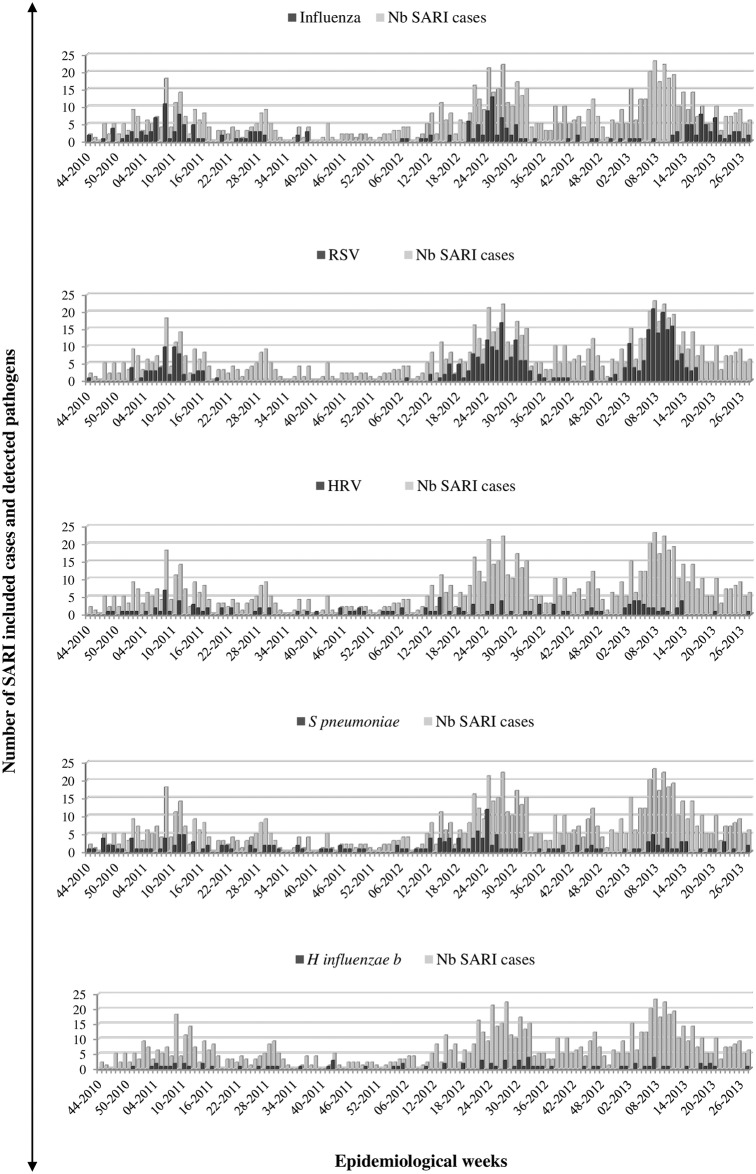
Weekly prevalence of the main respiratory pathogens detected among hospitalized SARI patients, Madagascar, November 2010 to July 2013. Each panel shows the weekly detection of one pathogen from SARI patients. For each pathogen, y-axis represents the weekly numbers of SARI and positive cases. RSV: Respiratory syncytial virus; RV: Rhinovirus; Hib: *Haemophilus influenzae* type b.

SARI cases were detected all year around during the 3 years of surveillance with distinct peaks of detection observed each year: between January and March in 2011 and 2013 and between May and July in 2012 (Fig 1). These peaks coincided with an active circulation of RSV and influenza viruses. Nevertheless, peaks of SARI appear to be more correlated to RSV circulation. Unlike other respiratory pathogens that are detected all year along, RSV seems to circulate once a year and being responsible of an epidemic every year.

## Discussion

To the best of our knowledge, this study is the first description of etiologies associated to hospitalized SARI patients from both urban and peri-urban areas in Madagascar. During the 3 years of surveillance, 867 consented patients meeting the established case definition of SARI were enrolled. The observed different clinical spectrum across age groups may be helpful to avoid misclassification of patients presenting with respiratory illness at the triage level when no standardized protocol is available.

The finding that about 82% of inpatients were tested positive for a pathogen is consistent with those reported elsewhere which showed etiologies ranging from 40% to 85% of hospitalized SARI cases depending on case definition or methodology design [14, 20, 21]. Most of the included patients were children less than 5 years old (78.5%). This study confirms that young children are the most vulnerable group to suffer from SARI [22, 23]. Nevertheless, these results could also be explained by a socio-economic behavior of the Malagasy population. Indeed, parents are keener to seek care for their children while older individuals try to cure themselves at home using self-medication or traditional medicine.

In the present study, about 43% of inpatients presented multiple infections by at least 2 pathogens. Here, viral infections were commonly found in 75.4% of tested patients which is similar to what was observed within the community during the influenza-like illness surveillance [19]. Taken together, the present study clearly demonstrates that respiratory viruses are the leading cause of severe acute respiratory illnesses in Madagascar. Overall, RSV was the most commonly detected followed by *S*. *pneumoniae* and influenza viruses.

Similar to other studies, substantial rate of RSV infection was observed in young population [14, 24, 25]. A meta-analysis of data from Africa reported that the incidence of RSV in lower acute respiratory infections that required hospitalization ranged from 10–18 per 1000 person year for infants and 3–9 per 1000 person year for children under 5 years of age [26]. The increased circulation of RSV that coincided with the peak of SARI cases may be informative for clinicians to predict an epidemic due to RSV and to choose the most appropriate care for patients in order to avoid overuse of antibiotics. In addition, the epidemic pattern of this virus reveals the need to develop rapid diagnostic tests to rapidly manage patients and reduce the number of hospitalization, and to promote effective vaccine to prevent severe infections in high-risk populations.

The role of influenza viruses in SARI-associated hospitalization is increasingly defined because of available data from active influenza sentinel surveillance system initiated in several countries including sub-Saharan Africa [27–30]. The proportion of influenza viruses in young population obtained here (20.4%) is much higher than in other African countries were SARI attributable to influenza are around 7% [16]. As seen elsewhere, the highest rate of hospitalization is obtained with the subtype A/H3N2 virus during its circulation [31, 32]. In Madagascar despite decades of reliable influenza surveillance, no national policy is implemented regarding vaccination. Moreover, influenza vaccines are not widely accessible and affordable for the whole population.

In the present study, about 35.2% of SARI cases were likely due to bacterial infection. Nevertheless, it is not clear what this high proportion of bacteria means due to the notion of carriage. The substantial rate of *S*. *pneumoniae* detected might be partly explained by the very low pneumococcal vaccination rate among the younger population (estimated at 0.4%) because most of the positive cases were detected before the introduction of pneumococcal vaccine into the expanded program on immunization (EPI) in Madagascar in 2012 [33]. This vaccine is believed to reduce substantially the burden of pneumococcal disease. Thus, it would be interesting to evaluate the proportion of SARI associated to *S*. *pneumoniae* more than 5 years after the introduction of the vaccine in Madagascar. On the other hand, the role of atypical bacteria in hospitalized ARI is poorly understood. One assumes that certain bacteria predispose to bacterial super-infections [34].

While RV is considered as the common cold virus, the present study showed that this virus accounted for 14% of total SARI cases. This finding highlights the potential role of RV in public health like other authors already postulated [35–37].

Our study presents some limitations. First, data were collected only from 2 sites that are not representative of the whole population preventing us to real estimate the prevalence at the national level. Indeed, prevalence of each pathogen may differ for regions having different bioclimatic or demographic patterns, access to healthcare, and connectivity. Since children are more at risk to develop severe infection, our sampling was skewed toward the younger group and likely underestimate the prevalence in adult. Indeed, few adult cases had been identified at the adult ward mainly because the duration of symptoms prior to admission exceeded the 7 days limit of our SARI case definition. It was also observed that severe cases were treated on external consultation or admitted at the emergency department for a few hours and then discharged. Patients that did not recover, worsened at home, and returned to the hospital were then admitted resulting in prolonged duration of illness (usually more than 7 days) at the time of admission. Another limitation is to attribute the pathogen(s) that is (are) the main cause that lead to severe illness. Indeed, we could not exclude that some pathogens may be present due to carriage.

## Conclusions

To conclude, the high prevalence of etiology associated to severe infections of relevant pathogens highlights the importance of sustaining national surveillance of SARI to clearly estimate the role of associated pathogens and establish the burden of disease. Further challenges in such program include efforts to implement strategies that capture adult cases as it is relevant to measure the impact of SARI in this population. Since vaccines play an important role in preventing SARI, vaccination against important pathogens, including RSV, should be accessible at least for high-risk population in developing countries. Indeed, although use of synthetic antibodies against RSV can be provided to certain at-risk infants, this treatment only provides a short-term protection, is not always effective, and is also expensive, putting it beyond the reach of developing countries like Madagascar.

## Supporting information

S1 Table Clinical diagnosis of patients hospitalized for SARI, Madagascar, November 2010 to July 2013. * LRTI: Low respiratory tract infection including bronchoalveolitis/exacerbation of chronic obstructive pulmonary disease (COPD), pleuropneumonia, and acute lobar pneumonia; ** other: neonatal infection, asthma, laryngitis, influenza-like illness etc… N = number of included patients. n = number of patients that responded by “yes” or “no” for a given symptom. Only bronchiolitis, pneumonia, and bronchopneumonia were statistically analyzed. Statistical analyses were performed using Fisher’s exact test.
(DOCX)

S2 Table Univariate analysis of the distribution of monoinfection and multiple infection detected in SARI adjusted by age groups, November 2010 to July 2013. The age group less than 5 years was considered as reference group.
(DOCX)

S3 Table Distribution of pathogens detected in patients hospitalized for SARI, November 2010 to July 2013. FLUA: Influenza virus A; FLUB: Influenza virus B; COV: Coronavirus; RSV: Respiratory syncytial virus; HMPV: Human metapneumovirus; PIV: Parainfluenza virus; *S*. *pneumoniae*: *Streptococcus pneumoniae*; Hib: *Haemophilus influenzae* type b; Staph: Staphylococcus; E: Escherichia; P: Pseudomonas. * Other species of Streptococcus. Statistical analyses were performed using Fisher’s exact test.
(DOCX)

S4 Table Results of logistic regression for demographic and clinical characteristics of SARI patients adjusted by age group, November 2010 to July 2013. GPD: General physical deterioration; MNW: Movement of nose wings; Inter recess: Intercostal recession. Statistical analyses were performed using Wald test. The age group less than 5 years was considered as reference group.
(DOCX)

## References

[1] Rudan I, Boschi-Pinto C, Biloglav Z, Mulholland K, and Campbell H. Epidemiology and etiology of childhood pneumonia. Bull World Health Organ. 2008; 86(5): p. 408–416. 10.2471/BLT.07.048769 18545744 10.2471/BLT.07.048769 PMC2647437

[2] WHO. The global burden of disease: 2004 update. Geneva: World Health Organization. 2008. http://www.who.int/healthinfo/global_burden_disease/GBD_report_2004update_full.pdf.

[3] Nair H, Simoes EA, Rudan I, Gessner BD, Azziz-Baumgartner E, Zhang JS, et al Global and regional burden of hospital admissions for severe acute lower respiratory infections in young children in 2010: a systematic analysis. Lancet. 2013; 381(9875): p. 1380–1390. 10.1016/S0140-6736(12)61901-1 23369797 10.1016/S0140-6736(12)61901-1 PMC3986472

[4] Rudan I, Tomaskovic L, Boschi-Pinto C, and Campbell H. Global estimate of the incidence of clinical pneumonia among children under five years of age. Bull World Health Organ. 2004; 82(12): p. 895–903. 15654403 PMC2623105

[5] Black RE, Cousens S, Johnson HL, Lawn JE, Rudan I, Bassani DG, et al Global, regional, and national causes of child mortality in 2008: a systematic analysis. Lancet. 2010; 375(9730): p. 1969–1987. 10.1016/S0140-6736(10)60549-1 20466419 10.1016/S0140-6736(10)60549-1

[6] Williams BG, Gouws E, Boschi-Pinto C, Bryce J, and Dye C. Estimates of world-wide distribution of child deaths from acute respiratory infections. Lancet Infect Dis. 2002; 2(1): p. 25–32. 10.1016/S1473-3099(01)00170-0 11892493 10.1016/s1473-3099(01)00170-0

[7] Watt JP, Wolfson LJ, O’Brien KL, Henkle E, Deloria-Knoll M, McCall N, et al Burden of disease caused by Haemophilus influenzae type b in children younger than 5 years: global estimates. Lancet. 2009; 374(9693): p. 903–911. 10.1016/S0140-6736(09)61203-4 19748399 10.1016/S0140-6736(09)61203-4

[8] O’Brien KL, Wolfson LJ, Watt JP, Henkle E, Deloria-Knoll M, McCall N, et al Burden of disease caused by Streptococcus pneumoniae in children younger than 5 years: global estimates. Lancet. 2009; 374(9693): p. 893–902. 10.1016/S0140-6736(09)61204-6 19748398 10.1016/S0140-6736(09)61204-6

[9] McIntosh K. Community-acquired pneumonia in children. N Engl J Med. 2002; 346(6): p. 429–437. 10.1056/NEJMra011994 11832532 10.1056/NEJMra011994

[10] Ruuskanen O and Mertsola J. Childhood community-acquired pneumonia. Semin Respir Infect. 1999; 14(2): p. 163–172. 10391410

[11] Choi EH, Lee HJ, Kim SJ, Eun BW, Kim NH, Lee JA, et al The association of newly identified respiratory viruses with lower respiratory tract infections in Korean children, 2000–2005. Clin Infect Dis. 2006; 43(5): p. 585–592. 10.1086/506350 16886150 10.1086/506350 PMC7107986

[12] Lim WS, Macfarlane JT, Boswell TC, Harrison TG, Rose D, Leinonen M, et al Study of community acquired pneumonia aetiology (SCAPA) in adults admitted to hospital: implications for management guidelines. Thorax. 2001; 56(4): p. 296–301. 10.1136/thorax.56.4.296 11254821 10.1136/thorax.56.4.296 PMC1746017

[13] Opatowski L, Fraser C, Griffin J, de Silva E, Van Kerkhove MD, Lyons EJ, et al Transmission characteristics of the 2009 H1N1 influenza pandemic: comparison of 8 Southern hemisphere countries. PLoS Pathog. 2011; 7(9): p. e1002225 10.1371/journal.ppat.1002225 21909272 10.1371/journal.ppat.1002225 PMC3164643

[14] Breiman RF, Cosmas L, Njenga M, Williamson J, Mott JA, Katz MA, et al Severe acute respiratory infection in children in a densely populated urban slum in Kenya, 2007–2011. BMC Infect Dis. 2015; 15: p. 95–106. 10.1186/s12879-015-0827-x 25879805 10.1186/s12879-015-0827-x PMC4351931

[15] Feikin DR, Njenga MK, Bigogo G, Aura B, Aol G, Audi A, et al Etiology and Incidence of viral and bacterial acute respiratory illness among older children and adults in rural western Kenya, 2007–2010. PLoS One. 2012; 7(8): p. e43656 10.1371/journal.pone.0043656 22937071 10.1371/journal.pone.0043656 PMC3427162

[16] McMorrow ML, Wemakoy EO, Tshilobo JK, Emukule GO, Mott JA, Njuguna H, et al Severe Acute Respiratory Illness Deaths in Sub-Saharan Africa and the Role of Influenza: A Case Series From 8 Countries. J Infect Dis. 2015; 212(6): p. 853–860. 10.1093/infdis/jiv100 25712970 10.1093/infdis/jiv100 PMC4826902

[17] Rajatonirina S, Rakotosolofo B, Rakotomanana F, Randrianasolo L, Ratsitoharina M, Raharinandrasana H, et al Excess mortality associated with the 2009 A(H1N1)v influenza pandemic in Antananarivo, Madagascar. Epidemiol Infect. 2013: p. 1–6. 10.1017/S0950268812001215 22814442 10.1017/S0950268812001215 PMC9151851

[18] Rajatonirina S, Razanajatovo NH, Ratsima EH, Orelle A, Ratovoson R, Andrianirina ZZ, et al Outcome Risk Factors during Respiratory Infections in a Paediatric Ward in Antananarivo, Madagascar 2010–2012. PLoS One. 2013; 8(9): p. e72839 10.1371/journal.pone.0072839 24069161 10.1371/journal.pone.0072839 PMC3771918

[19] Razanajatovo NH, Richard V, Hoffmann J, Reynes JM, Razafitrimo GM, Randremanana RV, et al Viral etiology of influenza-like illnesses in Antananarivo, Madagascar, July 2008 to June 2009. PLoS One. 2011; 6(3): p. e17579 10.1371/journal.pone.0017579 21390235 10.1371/journal.pone.0017579 PMC3048401

[20] Juven T, Mertsola J, Waris M, Leinonen M, Meurman O, Roivainen M, et al Etiology of community-acquired pneumonia in 254 hospitalized children. Pediatr Infect Dis J. 2000; 19(4): p. 293–298. 10783017 10.1097/00006454-200004000-00006

[21] Pretorius MA, Madhi SA, Cohen C, Naidoo D, Groome M, Moyes J, et al Respiratory viral coinfections identified by a 10-plex real-time reverse-transcription polymerase chain reaction assay in patients hospitalized with severe acute respiratory illness—South Africa, 2009–2010. J Infect Dis. 2012; 206 Suppl 1: p. S159–165. 10.1093/infdis/jis538 23169964 10.1093/infdis/jis538

[22] Cebey-Lopez M, Herberg J, Pardo-Seco J, Gomez-Carballa A, Martinon-Torres N, Salas A, et al Viral Co-Infections in Pediatric Patients Hospitalized with Lower Tract Acute Respiratory Infections. PLoS One. 2015; 10(9): p. e0136526 10.1371/journal.pone.0136526 26332375 10.1371/journal.pone.0136526 PMC4558027

[23] Hoffmann J, Rabezanahary H, Randriamarotia M, Ratsimbasoa A, Najjar J, Vernet G, et al Viral and atypical bacterial etiology of acute respiratory infections in children under 5 years old living in a rural tropical area of Madagascar. PLoS One. 2012; 7(8): p. e43666 10.1371/journal.pone.0043666 22912897 10.1371/journal.pone.0043666 PMC3422262

[24] Berkley JA, Munywoki P, Ngama M, Kazungu S, Abwao J, Bett A, et al Viral etiology of severe pneumonia among Kenyan infants and children. Jama. 2010; 303(20): p. 2051–2057. 10.1001/jama.2010.675 20501927 10.1001/jama.2010.675 PMC2968755

[25] Simpson MD, Kieke BA Jr., Sundaram ME, McClure DL, Meece JK, Sifakis F, et al Incidence of Medically Attended Respiratory Syncytial Virus and Influenza Illnesses in Children 6–59 Months Old During Four Seasons. Open Forum Infect Dis. 2016; 3(2): p. ofw081. 10.1093/ofid/ofw081 27419158 10.1093/ofid/ofw081 PMC4943552

[26] Nair H, Nokes DJ, Gessner BD, Dherani M, Madhi SA, Singleton RJ, et al Global burden of acute lower respiratory infections due to respiratory syncytial virus in young children: a systematic review and meta-analysis. Lancet. 2010; 375(9725): p. 1545–1555. 10.1016/S0140-6736(10)60206-1 20399493 10.1016/S0140-6736(10)60206-1 PMC2864404

[27] Kenmoe S, Tchendjou P, Vernet MA, Moyo-Tetang S, Mossus T, Njankouo-Ripa M, et al Viral etiology of severe acute respiratory infections in hospitalized children in Cameroon, 2011–2013. Influenza and Other Respiratory Viruses. 2016; 10(5): p. 386–393. 10.1111/irv.12391 27012372 10.1111/irv.12391 PMC4947949

[28] Randrianasolo L, Raoelina Y, Ratsitorahina M, Ravolomanana L, Andriamandimby S, Heraud JM, et al Sentinel surveillance system for early outbreak detection in Madagascar. BMC Public Health. 2010; 10: p. 31–39. 10.1186/1471-2458-10-31 20092624 10.1186/1471-2458-10-31 PMC2823701

[29] Cummings MJ, Bakamutumaho B, and O’Donnell MR. Unfinished business: severe acute respiratory infection in sub-Saharan Africa. Intensive Care Med. 2016; 42: p. 1515–1516. 10.1007/s00134-016-4383-7 27172822 10.1007/s00134-016-4383-7

[30] Nyamusore J, Rukelibuga J, Mutagoma M, Muhire A, Kabanda A, Williams T, et al The national burden of influenza-associated severe acute respiratory illness hospitalization in Rwanda, 2012–2014. Influenza and Other Respiratory Viruses. 2017: p. 1–8. 10.1111/irv.12494 29197152 10.1111/irv.12494 PMC5818355

[31] Simonsen L, Clarke MJ, Williamson GD, Stroup DF, Arden NH, and Schonberger LB. The impact of influenza epidemics on mortality: introducing a severity index. Am J Public Health. 1997; 87(12): p. 1944–1950. 9431281 10.2105/ajph.87.12.1944 PMC1381234

[32] Thompson WW, Shay DK, Weintraub E, Brammer L, Bridges CB, Cox NJ, et al Influenza-associated hospitalizations in the United States. Jama. 2004; 292(11): p. 1333–1340. 10.1001/jama.292.11.1333 15367555 10.1001/jama.292.11.1333

[33] Madagate. Unicef Madagascar: Introduction du vaccin contre le pneumocoque. Madagate.com. 2012. http://www.madagate.org/editorial/madagate-video-et-affiche/2737-unicef-madagascar-introduction-du-vaccin-contre-le-pneumocoque.html.

[34] Bellos A, Mulholland K, O’Brien KL, Qazi SA, Gayer M, and Checchi F. The burden of acute respiratory infections in crisis-affected populations: a systematic review. Confl Health. 2010; 4: p. 3–15. 10.1186/1752-1505-4-3 20181220 10.1186/1752-1505-4-3 PMC2829474

[35] Brini I, Guerrero A, Hannachi N, Bouguila J, Orth-Holler D, Bouhlel A, et al Epidemiology and clinical profile of pathogens responsible for the hospitalization of children in Sousse area, Tunisia. PLoS One. 2017; 12(11). 10.1371/journal.pone.0188325 29149199 10.1371/journal.pone.0188325 PMC5693464

[36] Martin ET, Fairchok MP, Stednick ZJ, Kuypers J, and Englund JA. Epidemiology of Multiple Respiratory Viruses in Childcare Attendees. The Journal of Infectious Diseases. 2013; 207: p. 982–989. 10.1093/infdis/jis934 23288925 10.1093/infdis/jis934 PMC7107308

[37] Pretorius M, Tempia S, Walaza S, Cohen AL, Moyes J, Variava E, et al The role of influenza, RSV and other common respiratory viruses insevere acute respiratory infections and influenza-like illness in apopulation with a high HIV sero-prevalence, South Africa 2012–2015. Journal of Clinical Virology. 2016; 75: p. 21–26. 26741826 10.1016/j.jcv.2015.12.004 PMC5712432

