# Supplementary material for: Epidemiology of severe acute respiratory infections from hospital-based surveillance in Madagascar, November 2010 to July 2013
Source: PLoS One. 2018 Nov 21;13(11):e0205124. doi: 10.1371/journal.pone.0205124 (PMC6248916; doi:10.1371/journal.pone.0205124)
Supplement: S1 Table — * LRTI: Low respiratory tract infection including bronchoalveolitis/exacerbation of chronic obstructive pulmonary disease (COPD), pleuropneumonia, and acute lobar pneumonia; ** other: neonatal infection, asthma, laryngitis, influenza-like illness etc… N = number of included patients. n = number of patients that responded by “yes” or “no” for a given symptom. Only bronchiolitis, pneumonia, and bronchopneumonia were statistically analyzed. Statistical analyses were performed using Fisher’s exact test. (DOCX) [file pone.0205124.s001.docx]

**S1 Table. Clinical diagnosis of patients hospitalized for SARI, Madagascar, November 2010 to July 2013.**

| **Variables** |  | **Bronchiolitis** | **Pneumonia** | **Bronchopneumonia** | **Other LRTI*** | **Other**** |
| --- | --- | --- | --- | --- | --- | --- |
|  | **n** | ***Presence (%)*** | ***Presence (%)*** | ***Presence (%)*** | ***Presence (%)*** | ***Presence (%)*** |
| **Global (N=876)** | 860 | 383 (44.5) | 76 (8.8) | 129 (15.0) | 127 (14.8) | 161 (18.7) |
| **Sites** |  |  |  |  |  |  |
| Antananarivo (N=657) | 649 | 285 (43.9) | 54 (8.3) | 115 (17.7) | 93 (14.3) | 102 (15.7) |
| Moramanga (N=219) | 211 | 98 (46.4) | 22 (10.4) | 14 (6.6) | 34 (16.1) | 51 (24.4) |
| *p-value* |  | 0.7 | 0.4 | <0.001 |  |  |
| **Age groups** |  |  |  |  |  |  |
| < 5yrs (N=710) |  | 366 (51.5) | 45 (6.3) | 66 (9.3) | 100 (14.1) | 133 (18.7) |
| 5-14yrs (N=37) |  | 12 (32.4) | 2 (5.4) | 7 (18.9) | 10 (27.0) | 6 (16.2) |
| 15-29yrs (N=26) |  | 1 (3.8) | 5 (19.2) | 13 (50.0) | 5 (19.2) | 2 (7.7) |
| 30-64yrs (N=84) |  | 4 (4.8) | 21 (25.0) | 34 (40.5) | 9 (10.7) | 16 (19.0) |
| >= 65yrs (N=19) |  | 0 (0.0) | 3 (15.8) | 9 (47.4) | 3 (15.8) | 4 (21.1) |
| *p-value* |  | <0.001 | <0.001 | <0.001 |  |  |

* LRTI: Low respiratory tract infection including bronchoalveolitis/exacerbation of chronic obstructive pulmonary disease (COPD), pleuropneumonia, and acute lobar pneumonia; ** other: neonatal infection, asthma, laryngitis, influenza-like illness etc…

N=number of included patients.

n=number of patients that responded by “yes” or “no” for a given symptom.

Only bronchiolitis, pneumonia, and bronchopneumonia were statistically analyzed. Statistical analyses were performed using Fisher’s exact test.
