# Supplementary material for: Epidemiology of severe acute respiratory infections from hospital-based surveillance in Madagascar, November 2010 to July 2013
Source: PLoS One. 2018 Nov 21;13(11):e0205124. doi: 10.1371/journal.pone.0205124 (PMC6248916; doi:10.1371/journal.pone.0205124)
Supplement: S2 Table — The age group less than 5 years was considered as reference group. (DOCX) [file pone.0205124.s002.docx]

**S2 Table : Univariate analysis of the distribution of monoinfection and multiple infection detected in SARI by age group, November 2010 to July 2013.**

| **Age groups** | **Global N=876** | **Positive N=728** | **Monoinfection N=340** | **Multiple infection N=388** | **OR [95%CI]** | **p-value** |
| --- | --- | --- | --- | --- | --- | --- |
| < 5yrs | 710 (81.1%) | 605 (83.1%) | 269 (79.1%) | 336 (86.6%) |  |  |
| 5-14yrs | 37 (4.2%) | 33 (4.5%) | 15 (4.4%) | 18 (4.6%) | 1 [0.5-1.9] | 0.9 |
| 15-29yrs | 26 (3.0%) | 17 (2.3%) | 10 (2.9%) | 7 (1.8%) | 0.5 [0.2-1.5] | 0.2 |
| 30-64yrs | 84 (9.6%) | 58 (8.0%) | 38 (11.2%) | 20 (5.2%) | 0.4 [0.2-0.7] | 0.003 |
| >= 65yrs | 19 (2.2) | 15 (2.1%) | 8 (2.4%) | 7 (1.8%) | 0.7 [0.3-2.0] | 0.5 |

The age group less than 5 years was considered as reference group.
