# Supplementary material for: Epidemiology of severe acute respiratory infections from hospital-based surveillance in Madagascar, November 2010 to July 2013
Source: PLoS One. 2018 Nov 21;13(11):e0205124. doi: 10.1371/journal.pone.0205124 (PMC6248916; doi:10.1371/journal.pone.0205124)
Supplement: S3 Table — FLUA: Influenza virus A; FLUB: Influenza virus B; COV: Coronavirus; RSV: Respiratory syncytial virus; HMPV: Human metapneumovirus; PIV: Parainfluenza virus; S. pneumoniae: Streptococcus pneumoniae; Hib: Haemophilus influenzae type b; Staph: Staphylococcus; E: Escherichia; P: Pseudomonas. * Other species of Streptococcus. Statistical analyses were performed using Fisher’s exact test. (DOCX) [file pone.0205124.s003.docx]

**S3 Table. Distribution of pathogens detected in patients hospitalized for SARI, November 2010 to July 2013.**

|  | **Global**  **(N=876)** | **Antananarivo**  **(N=657)** | **Moramanga (N=219)** | **p-value** |
| --- | --- | --- | --- | --- |
| **Pathogens** | ***Presence (%)*** | ***Presence (%)*** | ***Presence (%)*** |  |
| FLUA | 170 (19.4) | 138 (21.0) | 32 (14.6) | 0.039 |
| FLUB | 58 (6.6) | 45 (6.8) | 13 (5.9) | 0.754 |
| COV-OC43 | 21 (2.4) | 14 (2.1) | 7 (3.2) | 0.443 |
| COV-NL63 | 15 (1.7) | 12 (1.8) | 3 (1.4) | 0.773 |
| COV-229E | 4 (0.5) | 2 (0.3) | 2 (0.9) | 0.261 |
| COV-HKU1 | 2 (0.2) | 2 (0.3) | 0 (0.0) | --- |
| RSV | 348 (39.7) | 265 (40.3) | 83 (37.9) | 0.577 |
| HMPV | 33 (3.8) | 28 (4.3) | 5 (2.3) | 0.222 |
| Rhinovirus | 125 (14.3) | 91 (13.9) | 34 (15.5) | 0.577 |
| PIV-1 | 10 (1.1) | 7 (1.1) | 3 (1.4) | 0.717 |
| PIV-2 | 12 (1.4) | 10 (1.5) | 2 (0.9) | 0.740 |
| PIV-3 | 9 (1.0) | 6 (0.9) | 3 (1.4) | 0.698 |
| Adenovirus | 77 (8.8) | 55 (8.4) | 22 (10.0) | 0.491 |
| Bocavirus | 40 (4.6) | 31 (4.7) | 9 (4.1) | 0.852 |
| *S. pneumoniae* | 186 (21.2) | 155 (23.6) | 31 (14.2) | 0.003 |
| Hib | 79 (9.0) | 67 (10.2) | 12 (5.5) | 0.040 |
| Streptococcus* | 30 (3.4) | 24 (3.7) | 6 (2.7) | 0.669 |
| Klebsiella | 17 (1.9) | 11 (1.7) | 6 (2.7) | 0.3 |
| Branhamella | 15 (1.7) | 13 (2.0) | 2 (0.9) | 0.6 |
| Enterobacter | 14 (1.6) | 11 (1.7) | 3 (1.4) | 0.9 |
| *Staph aureus* | 9 (1.0) | 7 (1.1) | 2 (0.9) | 0.8 |
| *E. coli* | 4 (0.5) | 3 (0.5) | 1 (0.5) | 0.3 |
| Acinetobacter | 3 (0.3) | 3 (0.5) | 0 (0.0) | --- |
| Aerococcus | 3 (0.3) | 3 (0.5) | 0 (0.0) | --- |
| Moraxella | 2 (0.2) | 2 (0.3) | 0 (0.0) | --- |
| *P. aeruginosa* | 2 (0.2) | 1 (0.2) | 1 (0.5) | --- |
| Listeria | 1 (0.1) | 1 (0.2) | 0 (0.0) | --- |
| *Staph haemolyticus* | 1 (0.1) | 1 (0.2) | 0 (0.0) | --- |
| Stenotrophomonas | 1 (0.1) | 1 (0.2) | 0 (0.0) | --- |
| *Proteus mirabilis* | 1 (0.1) | 1 (0.2) | 0 (0.0) | --- |
| *Serratia marcescens* | 1 (0.1) | 1 (0.2) | 0 (0.0) | --- |

FLUA: Influenza virus A; FLUB : Influenza virus B ; COV: Coronavirus; RSV: Respiratory syncytial virus; HMPV: Human metapneumovirus; PIV: Parainfluenza virus; *S. pneumoniae*: *Streptococcus pneumoniae*; Hib: *Haemophilus influenzae* type b; Staph: Staphylococcus; E: Escherichia; P: Pseudomonas. * Other species of Streptococcus. Statistical analyses were performed using Fisher’s exact test.
