# Supplementary material for: Epidemiology of severe acute respiratory infections from hospital-based surveillance in Madagascar, November 2010 to July 2013
Source: PLoS One. 2018 Nov 21;13(11):e0205124. doi: 10.1371/journal.pone.0205124 (PMC6248916; doi:10.1371/journal.pone.0205124)
Supplement: S4 Table — GPD: General physical deterioration; MNW: Movement of nose wings; Inter recess: Intercostal recession. Statistical analyses were performed using Wald test. The age group less than 5 years was considered as reference group. (DOCX) [file pone.0205124.s004.docx]

**S4 Table. Results of logistic regression for demographic and clinical characteristics of SARI patients adjusted by age group, November 2010 to July 2013.**

|  |  | **< 5yrs (N=710)** | **5-14yrs**  **(N=37)** | **15-29yrs (N=26)** | **30-64yrs (N=84)** | **>= 65yrs (N=19)** |
| --- | --- | --- | --- | --- | --- | --- |
| **Gender** | **N=876** | **n (%)** | **n (%)** | **n (%)** | **n (%)** | **n (%)** |
| Female | *396* | 325 (82.1) | 17 (4.3) | 11 (2.8) | 34 (8.6) | 9 (2.3) |
| Male | *480* | 385 (80.2) | 20 (4.2) | 15 (3.1) | 50 (10.4) | 10 (2.1) |
|  | *OR [95%CI]* | 1 | 0.99 [0.5-1.9] | 1.2 [0.5-2.6] | 1.2 [0.8-2.0] | 0.9 [0.4-2.4] |
|  | *p-value* | -- | 0.9 | 0.7 | 0.4 | 0.9 |
| **Symptoms** |  |  |  |  |  |  |
| Fever | ***860*** | 424 (61.1) | 37 (100.0) | 26 (100.0) | 84 (100.0) | 19 (100.0) |
| Dry cough | ***857*** | 321 (46.4) | 14 (37.8) | 15 (60.0) | 46 (54.8) | 15 (78.9) |
|  | *OR [95%CI]* | 1 | 0.7 [0.4-1.4] | 1.7 [0.8-4.0] | 1.4 [0.9-2.2] | 4.3 [1.6-15.3] |
|  | *p-value* | -- | 0.3 | 0.2 | 0.1 | 0.01 |
| Productive cough | ***857*** | 363 (52.5) | 24 (64.9) | 12 (46.2) | 44 (52.4) | 11 (57.9) |
|  | *OR [95%CI]* | 1 | 1.7 [0.8-3.4] | 0.8 [0.3-1.7] | 0.99 [0.3-1.7] | 1.2 [0.5-3.2] |
|  | *p-value* | -- | 0.1 | 0.5 | 1 | 0.6 |
| Dyspnea | ***860*** | 585 (84.3) | 32 (86.5) | 23 (88.5) | 80 (95.2) | 17 (89.5) |
|  | *OR [95%CI]* | 1 | 1.2 [0.5-3.6] | 1.4 [0.5-6.1] | 3.7 [1.5-12.4] | 1.6 [0.5-10.1] |
|  | *p-value* | -- | 0.7 | 0.6 | 0.01 | 0.5 |
| Chest pain | ***751*** | 43 (7.3) | 8 (22.2) | 9 (34.6) | 42 (50.6) | 8 (42.1) |
|  | *OR [95%CI]* | 1 | 3.6 [1.5-8.1] | 6.7 [2.7-15.6] | 13 [7.6-22.2] | 9.2 [3.4-24.0] |
|  | *p-value* | -- | 0.003 | <0.001 | <0.001 | <0.001 |
| Runny nose | ***858*** | 524 (75.7) | 30 (81.1) | 7 (26.9) | 30 (35.7) | 7 (36.8) |
|  | *OR [95%CI]* | 1 | 1.4 [0.6-3.5] | 0.1 [0.1-0.3] | 0.2 [0.1-0.3] | 0.2 [0.1-0.5] |
|  | *p-value* | -- | 0.459 | <0.001 | <0.001 | 0.001 |
| Sore throat | ***783*** | 58 (9.4) | 10 (27.0) | 7 (26.9) | 16 (19.0) | 4 (21.1) |
|  | *OR [95%CI]* | 1 | 3.6 [1.6-7.5] | 3.6 [1.3-8.5] | 2.3 [1.2-4.1] | 2.6 [0.7-7.4] |
|  | *p-value* | -- | 0.001 | 0.006 | 0.008 | 0.1 |
| Headache | ***753*** | 32 (5.4) | 2 (5.7) | 13 (50.0) | 48 (57.8) | 12 (63.2) |
|  | *OR [95%CI]* | 1 | 1.1 [0.2-3.7] | 17.4 [7.4-41.1] | 23.9 [13.6-42.5] | 29.9 [11.3-85.3] |
|  | *p-value* | -- | 0.9 | <0.001 | <0.001 | <0.001 |
| Thrill | ***851*** | 62 (9.0) | 6 (16.7) | 15 (57.7) | 50 (59.5) | 12 (63.2) |
|  | *OR [95%CI]* | 1 | 2 [0.7-4.7] | 13.7 [6.1-31.9] | 14.8 [9.0-24.8] | 17.3 [6.7-47.9] |
|  | *p-value* | -- | 0.1 | <0.001 | <0.001 | <0.001 |
| Sweats | ***856*** | 161 (23.3) | 11 (29.7) | 19 (73.1) | 54 (64.3) | 14 (73.7) |
|  | *OR [95%CI]* | 1 | 1.4 [0.7-2.8] | 8.9 [3.8-23.2] | 5.9 [3.7-10.0] | 9.2 [3.5-28.8] |
|  | *p-value* | -- | 0.4 | <0.001 | <0.001 | <0.001 |
| Anorexia | ***859*** | 325 (46.9) | 20 (54.1) | 18 (69.2) | 52 (61.9) | 17 (89.5) |
|  | *OR [95%CI]* | 1 | 1.3 [0.7-2.6] | 2.6 [1.1-6.3] | 1.8 [1.2-3.0] | 9.6 [2.7-61.0] |
|  | *p-value* | -- | 0.4 | 0.03 | 0.01 | 0.003 |
| Vomiting | ***861*** | 162 (23.3) | 7 (18.9) | 4 (15.4) | 17 (20.2) | 1 (5.3) |
|  | *OR [95%CI]* | 1 | 0.8 [0.3-1.7] | 0.6 [0.8-1.6] | 0.8 [0.8-1.6] | 0.2 [0.01-0.9] |
|  | *p-value* | -- | 0.5 | 0.4 | 0.5 | 0.1 |
| Diarrhea | ***859*** | 90 (13.0) | 5 (13.5) | 2 (7.7) | 14 (16.7) | 1 (5.3) |
|  | *OR [95%CI]* | 1 | 1.1 [0.4-2.5] | 0.6 [0.1-1.9] | 1.3 [0.7-2.4] | 0.4 [0.02-1.8] |
|  | *p-value* | -- | 0.9 | 0.4 | 0.4 | 0.3 |
| Weight loss | ***853*** | 171 (24.9) | 12 (32.4) | 16 (61.5) | 34 (40.5) | 12 (63.2) |
|  | *OR [95%CI]* | 1 | 1.5 [0.7-2.9] | 4.8 [2.2-11.2] | 2.1 [1.3-3.3] | 5.2 [2.1-14.1] |
|  | *p-value* | -- | 0.3 | <0.001 | 0.003 | 0.001 |
| Asthenia | ***854*** | 302 (43.9) | 17 (45.9) | 21 (80.8) | 65 (77.4) | 17 (89.5) |
|  | *OR [95%CI]* | 1 | 1.1 [0.6-2.1] | 5.4 [2.2-16.2] | 4.4 [2.6-7.6] | 10.9 [3.1-68.9] |
|  | *p-value* | -- | 0.8 | 0.001 | <0.001 | 0.002 |
| GPD | ***859*** | 174 (25.1) | 7 (18.9) | 11 (42.3) | 22 (26.2) | 13 (68.4) |
|  | *OR [95%CI]* | 1 | 0.7 [0.3-1.5] | 2.2 [1.0-4.8] | 1.1 [0.6-1.8] | 6.5 [2.5-18.6] |
|  | *p-value* | -- | 0.4 | 0.05 | 0.8 | <0.001 |
| Inter recess | ***855*** | 516 (74.7) | 23 (62.2) | 12 (46.2) | 38 (46.3) | 9 (47.4) |
|  | *OR [95%CI]* | 1 | 0.6 [0.3-1.1] | 0.3 [0.1-0.6] | 0.3 [0.2-0.5] | 0.3 [0.1-0.8] |
|  | *p-value* | -- | 0.1 | 0.002 | <0.001 | 0.011 |
| MNW | ***854*** | 368 (53.4) | 17 (45.9) | 12 (46.2) | 41 (49.4) | 8 (42.1) |
|  | *OR [95%CI]* | 1 | 0.7 [0.4-1.4] | 0.8 [0.34-1.6] | 0.9 [0.5-1.3] | 0.6 [0.2-1.6] |
|  | *p-value* | -- | 0.4 | 0.5 | 0.5 | 0.3 |
| Cyanosis | ***848*** | 101 (14.7) | 3 (8.1) | 6 (23.1) | 26 (32.5) | 5 (26.3) |
|  | *OR [95%CI]* | 1 | 0.5 [0.1-1.5] | 1.7 [0.6-4.2] | 2.8 [1.7-4.6] | 2.1 [0.7-5.5] |
|  | *p-value* | -- | 0.3 | 0.2 | <0.001 | 0.2 |

GPD: General physical deterioration; Inter recess: Intercostal recession; MNW: Movement of nose wings. The age group less than 5 years was considered as reference group. Statistical analyses were performed using Wald test. The age group less than 5 years was considered as reference group.
